# Closed-Loop Elastic Demand Control under Dynamic Pricing Program in Smart Microgrid Using Super Twisting Sliding Mode Controller

**DOI:** 10.3390/s20164376

**Published:** 2020-08-05

**Authors:** Taimoor Ahmad Khan, Kalim Ullah, Ghulam Hafeez, Imran Khan, Azfar Khalid, Zeeshan Shafiq, Muhammad Usman, Abdul Baseer Qazi

**Affiliations:** 1 Department of Electrical Engineering, University of Engineering and Technology, Mardan 23200, Pakistan; taymourkahn@gmail.com (T.A.K.); kalimullahbtk1@gmail.com (K.U.); ghulamhafeez393@gmail.com (G.H.); imran@uetmardan.edu.pk (I.K.); zeeshan@uetmardan.edu.pk (Z.S.); 2 Department of Electrical and Computer Engineering, COMSATS University Islamabad, Islamabad 44000, Pakistan; 3 Department of Engineering, School of Science & Technology, Nottingham Trent University, Nottingham NG11 8NS, UK; 4 Department of Computer Software Engineering, University of Engineering and Technology, Mardan 23200, Pakistan; usman@uetmardan.edu.pk; 5 Department of Software Engineering, Bahria University, Islamabad 44000, Pakistan; abq.buic@bahria.edu.pk

**Keywords:** smart grid, microgrid, internet of things, sensors, demand response, elastic demand control, dynamic energy pricing, super twisting sliding mode controller

## Abstract

Electricity demand is rising due to industrialisation, population growth and economic development. To meet this rising electricity demand, towns are renovated by smart cities, where the internet of things enabled devices, communication technologies, dynamic pricing servers and renewable energy sources are integrated. Internet of things (IoT) refers to scenarios where network connectivity and computing capability is extended to objects, sensors and other items not normally considered computers. IoT allows these devices to generate, exchange and consume data without or with minimum human intervention. This integrated environment of smart cities maintains a balance between demand and supply. In this work, we proposed a closed-loop super twisting sliding mode controller (STSMC) to handle the uncertain and fluctuating load to maintain the balance between demand and supply persistently. Demand-side load management (DSLM) consists of agents-based demand response (DR) programs that are designed to control, change and shift the load usage pattern according to the price of the energy of a smart grid community. In smart grids, evolved DR programs are implemented which facilitate controlling of consumer demand by effective regulation services. The DSLM under price-based DR programs perform load shifting, peak clipping and valley filling to maintain the balance between demand and supply. We demonstrate a theoretical control approach for persistent demand control by dynamic price-based closed-loop STSMC. A renewable energy integrated microgrid scenario is discussed numerically to show that the demand of consumers can be controlled through STSMC, which regulates the electricity price to the DSLM agents of the smart grid community. The overall demand elasticity of the current study is represented by a first-order dynamic price generation model having a piece-wise linear price-based DR program. The simulation environment for this whole scenario is developed in MATLAB/Simulink. The simulations validate that the closed-loop price-based elastic demand control technique can trace down the generation of a renewable energy integrated microgrid.

## 1. Introduction

With industrialisation, population growth and economic development, the dependence on electricity is ever so increasing, and consequently, electricity consumption is on the hike. This increased electricity consumption causes a problem of energy scarcity and environmental degradation. The conventional grid is unable to solve such problems. Thus, smart grid is stimulated as a smart solution. The smart grid introduces novel ways of electricity generation, namely renewable energy. The smart cities in the smart grid accommodate renewable energy sources, the internet of things enabled devices, communication technologies and dynamic pricing servers to meet this rising electricity demand [1].

The electricity generation from renewable energy sources in smart cities employing internet of things enabled devices is drawing a lot of attention from researchers nowadays due to their environment-friendliness and sustainability. Moreover, due to the high electricity consumption of conventional devices and high operational costs of conventional generators, the internet of things enabled devices, and renewable energy generation is preferred [2]. Keeping in mind the advantages of renewable energy over fossil and radioactive fuel-based generation, renewable energy is integrated into microgrids to reduce dependence on the fossil fuel-based power grid, thereby, increasing energy efficiency. Carbon emissions (CO_2_) are expected to reduce due to renewable energy integrated microgrids [3]. The energy balance of the system becomes more challenging due to wind and solar energy unavailability throughout the year. Therefore, a lot of work is required to address the dependability on fluctuating renewable energy integrated microgrids. In order to meet the demand of the consumers, balance has to be maintained or consequently consumers will face short term outages. To optimally balance the demand with supply, electric load forecasting is mandatory [4]. By installing demand-side load management (DSLM) in smart meters at consumers’ premises, the system will get the demand response (DR) of a consumer which will help to adjust the energy demand with the fluctuating generation [5]. Researchers have also developed new techniques for two-way communication between consumer and supplier end in recent publications signifying closed-loop demand and generation control schemes.

By installing agents-based DSLM systems at the consumer end, consumer demand will become more predictable and deterministic in future smart grids [6,7]. Authors used classical controllers with heuristic approaches to control the frequency regulations for two area power systems [8]. However, the DR of the system is not considered. A sliding mode controller (SMC) is proposed for controlling of automatic generation of interconnected multi-area power systems for the deregulated scenario [9]. Multiple one inputs and outputs data-driven SMC problem of nonlinear discrete systems are presented [10], where authors used the non-parametric dynamic linearization technique and second-order sliding mode control law based on the proportional integration differential (PID) sliding surface to obtain much faster transient response and smaller steady-state tracking error. The chattering phenomenon is slightly reduced and also compared with other methods. Chattering is the phenomenon of finite frequency and finite amplitude oscillations found in the response of implementing sliding mode algorithm. Chattering is caused by the high frequency switching of the sliding mode controller exciting un-modulated dynamics in a closed-loop system. In addition, the optimal gains of SMC are determined through the gravitational search algorithm (GSA) [11]. Various literature are discussed on automatic generation control, however, demand control is not discussed by the authors [12]. An approach for tuning a feedback control system is presented [13], with a linear plant consisting of super twisting sliding mode controller (STSMC), which shows that chattering motion occurs when the linear plant is having greater than one degree. A closed-loop elastic demand control strategy based on dynamic pricing by proportional-integral (PI) controller was proposed [14], although the elastic demand is controlled after 5.5 h but the demand overshoots in the first 5.5 h after the start of simulations. This work contributes to the literature based on the identified research gaps, which is elaborated in the subsequent text.

In this study, we aim to control the elastic demand for future smart grids. For this, we discussed the problem of the elastic demand control and its automation through dynamic pricing and regulating it to the DR programs at the consumer’s end. Then, a brief discussion is conducted on opportunities of closed-loop elastic demand control by employing a dynamic price of demand response programs. In addition, the STSMC is employed to control a closed-loop elastic demand to minimise energy balance error using dynamic energy price of demand response programs. The operation of the proposed model is briefly explained as dependence on a mismatch between demand and supply called closed-loop feedback error (balance error) where STSMC creates the pricing signal and drives the feedback error towards 0 in time. According to the control theory, a closed-loop control system is stable, if its balance error approaches to 0. Thus, in our case, closed-loop elastic demand control based on STSMC obtains energy balance by persistently broadcasting energy prices.

For simulations, we have developed a model in MATLAB/Simulink environment, which is composed of STMC and a dynamic pricing demand response model with feedback. The demand response model developed in Simulink is based on a first-order dynamic system with variable gain. The developed model has applied on an example smart microgrid integrated with renewable energy to control closed-loop elastic demand control for demand-side management. The efficacy of the proposed STMC-based model is validated by comparing it to the benchmark models based on PI controller, FOPI controller, FOPID controller and FOPD controller in terms of objectives. Simulation results illustrate that our proposed STSMC based closed demand control is affective to maintain energy balance while generation fluctuating in smart microgrids.

The structure of the paper is as follows: Section 2 describes related work and in Section 3 existing and proposed methods are discussed. Section 4 discussed proposed architecture. In Section 5, simulation results and formulation of scenarios are demonstrated. Finally, the paper is concluded in Section 6.

## 2. Related Work

In the literature, various techniques are implemented by researchers in recent years to control the demand of consumers on price-based demand models. Price-based DR models are presented [15,16]. A robust optimisation approach is presented for setting the short-term dynamic retail rates for an 22 asset-light retail energy providers. With this approach, the REP can decide how to participate in forward contracts and call options. In addition, REP can determine the optimal operation of the self-generation DG units [17]. DR for the consumers can be easily implemented through smart meters to solve the unbalance in generation and demand [18]. Flexible load control is developed which is based on the consumer DR for regulations in generation [19]. The efficiency of DR increased [20] by dividing it into three DR programs, i.e., natural DR (nDR), mandatory DR (mDR) and emergency DR (eDR). In literature [21], the author used a binary backtracking search algorithm with ZigBee, smart sockets and binary particle swarm optimisation to reduce energy consumption and electricity cost in peak hours. Authors proposed a model to minimise 10.25% cost by shifting and scheduling of different loads based on price [22]. In addition, by multi-objective particle swarm optimisation (MOPSO), emissions are reduced by charging penalties based on time. By using the model predictive control with pseudocode (M) [23], authors use a battery storage system with solar panels to reduce cost during peak hours. Authors provide a solution which is based on load scheduling and hybrid switch controlling to provide sustainable energy to consumers [24]. It also increased system efficiency and reduced cost of electricity concerning the demand of the customers. Using real-time pricing the consumers are forced to shift its loads towards the wind energy generation to reduce operational cost and per unit electricity cost [25]. An EV user behaviour simulator is introduced, which works in combination with an innovative smart distribution, locational and marginal price based on operation, to understand the influence of the dynamic energy pricing on both demand and supply side. The purpose of doing so is to understand the affect of dynamic pricing on the operation of the smart grid and the electric vehicle (EV) charging cost [26]. Consumer inconvenience caused by DR programs is reduced by mixed-integer linear programming (MILP) algorithm [27]. Using DSLM with the concept of load shifting technique, the author reduced overall peak load demand [28]. By shifting and load scheduling using a fuzzy logic controller with energy management algorithm (EMA), renewable energy generation from wind is increased by 2.5% and solar is increased by 2%. In addition, the fuel cell fuel consumption is reduced up to 4% annually [29]. Based on the operational history of industrial air separation units, the author in [30] presents a dynamic optimisation-based DR scheduling framework. The authors in [31] minimise heating ventilation air conditioning (HVAC) power intake using model-predictive-control (MPC) in peak hours without causing thermal convenience to the consumer and optimise the scheduling of battery which results in the decrease of monthly electricity bill. In generation uncertainty, flexible demand plays an essential role in the improvement of energy demand following generation and thus balancing the energy to some extent [32]. The response of load from DSLM will make the demand of the consumers more predictable and deterministic in near time for smart grids which is based on closed-loop systems with techniques like mild modified intrusive genetic algorithm (MMIGA) and cloud based infrastructure (CBI) [33,34,35]. In smart meters, the agents of the DSLM system can decrease the electricity bills of consumers at peak hours [36]. Authors with the help of a home energy management system, optimise the energy consumed by the appliances in a smart home [37]. Appliances are classified based on its operation priority to the consumer as elastic, inelastic, controllable, uncontrollable, interruptible, uninterruptible, delayable, etc. [38,39,40,41,42]. The related work in terms of techniques, models, objectives, results, and limitations is summerized in Table 1.

## 3. Existing Work and Proposed System Model

The performance of the integral controller (I), PI controller, PID controller and integral derivative controller (ID) are discussed whose parameters are tuned through the genetic algorithm (GA) through which generation is controlled [9]. The results are taken separately and then compared in terms of undershooting, overshooting and settling time of the deviations. Results have been taken with SMC for the same scenario which is better than the classical controllers. The generation from multiple source generation and DR of the consumer are discussed [43]. Here, the fractional-order classical PI and PID controllers are used for tracing the generation by demand of consumers under uncertain conditions. Although the demand traced the generation, there is a clear gap between both of them. In addition, a PI controller [14] is used for balancing the error or the mismatch between the generation and the consumers’ demand. Consumers’ demand is adjusted according to generation. The gap between the demand and generation [43] has become narrower [14] but the demand traced the generation after an overshoot for the first 5.5 h. The STSMC is unquestionably robust and accurate when deployed for stabilising nonlinear-systems. The STSMC has attracted the researcher community and gained a considerable research interest in the area of control. The results of various types of SMC are discussed in detail in the literature [44]. The Lyapunov function is proposed for SMC for the case when it is affected by bounded external perturbations and estimated the global finite time of stability [45,46]. Additionally, a global non-smooth Lyapunov function is proposed for SMC which is based on the combination of global exponential and finite-time stability of switched systems [47]. Related discussions for the SMC on the similar topics can also be found [48,49,50]. A step by step SMC is used for an application in robotics and aerospace [51,52].

### 3.1. Overview of Fractional Order Proportional Integral (FOPI) Controller

**Figure 1:**
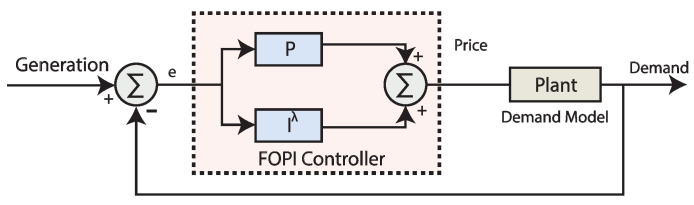
Block diagram of simulations using FOPI controller in closed-loop elastic demand control.

The replacement of the integral and derivative operators of the classical PI controller leads to a controller called fractional order PI controller. The application of real order for the integral and derivative operators is the significant foundation of fractional calculus. Numerous descriptions exist in the literature for the FO derivative, but an FOPI controller proposed for the closed-loop elastic demand control is employed [43]. In addition, for the other versions of classical controllers, replacing the operators for the integrals and derivative will lead to the formation of fractional order controller for the respective controller. Block diagram of using FOPI controller in simulations for closed-loop demand control is illustrated in Figure 1.

The transfer function for the version of FOPI controller commonly written as:

(1) $$C_{FOPI}\left(s\right)=K_{p}+\frac{K_{i}}{s^{\lambda }}$$

where, $K_{p}$ is the proportional gain coefficient, $K_{i}$ is the integral gain coefficient and the parameter λ is the fractional integrator order. A fourth order integer order (FOIO) approximation of FO integration by adopting the well known Continued Fractional Expansion (CFE) method [43].

### 3.2. Overview of Proportional Integral Derivative (PID) Controller

**Figure 2:**
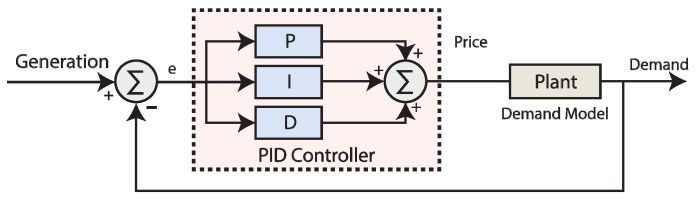
Block diagram of simulations using a proportional integral derivative (PID) controller in using closed-loop elastic demand control.

The PID control loop mechanism is the best possible solution for minimising the online and unknown faults in real time operations. Applications of the PID controller in industry is to access the single fine grained measurements for the improvement of the closed-loop operations. Tuning of the coefficients of PID controller is a challenging step for the researcher and in itself has a wide area of research. For simplicity of the controller, we used the coefficients’ values used for tuning the coefficients of the PID controller [43]. Block diagram of using PID controller in simulations for closed-loop demand control is illustrated in Figure 2.

(2) $$C\left(t\right)=K_{p}e\left(t\right)+K_{i}\int_{0}^{t}e(t)dt+K_{d}\frac{de(t)}{dt}$$

PIDs have 3 coefficients; $K_{p}$, $K_{i}$ and $K_{d}$. Where $K_{p}$ is the proportional gain constant, $K_{i}$ is the integral gain constant, $K_{d}$ is the derivative gain constant and e is the error defined as the difference between the set-point and the process variable value. The relation of I/O of an ideal relation for the PID controller in s domain is given as:

(3) $$C_{PID}\left(s\right)=K_{p}e+K_{i}e\frac{1}{s}+K_{d}es$$

### 3.3. Overview of Proportional Integral (PI) Controller

**Figure 3:**
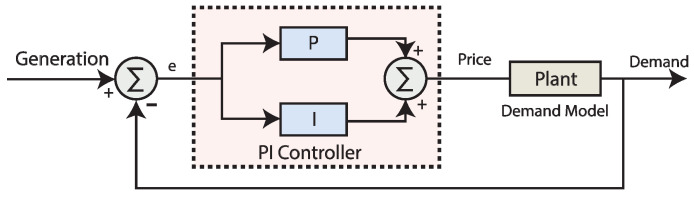
Block diagram of simulations using a proportional integral (PI) controller in closed-loop elastic demand control.

In closed-loop industrial processes, the combination of proportional and integral gains is the most widely used classical controller which plays vital role in balancing of level, flow, pressure and other such like industrial process variables that do not involve too much delays. While balancing the industrial plant problems, the PI controller still has problems that inherit with the controller actions. Usually there is high overshoot, initial value and a greater response time of using PI controller for a larger load demand. The author used a PI controller for elastic demand control [14]. There is an overshoot for the first 5.5 h in consumers’ demand which results in the lack of supply of energy at the consumers’ end. PI controller takes long stabilisation time with overshoot while dealing high variations in the system which effects the system stability at the start of process. A conventional PI controller has 2 gain coefficients, $K_{p}$ and $K_{i}$. $K_{p}$ is known as the proportional coefficient while $K_{i}$ is the integral coefficient of the PI controller. The relation of I/O of a PI controller can be written as:

(4) $$C_{PI}\left(s\right)=K_{p}e+K_{i}e\frac{1}{s}$$

where *e* is the error signal in negative feedback closed-loop system. Block diagram of using PI controller in simulations for closed-loop demand control is illustrated in Figure 3.

### 3.4. Overview of Fractional Order Proportional Derivative (FOPD) Controller

**Figure 4:**
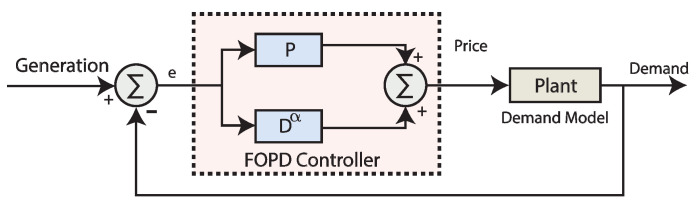
Block diagram of simulations using a fractional order proportional derivative (FOPD) controller in closed-loop elastic demand control.

A specific form of the most common fractional order PID control where I and fractional operator of I is equal to zero which forms a fractional order proportional derivative controller. The relation of I/O for the FOPD controller is commonly written as;

(5) $$C_{FOPD}\left(s\right)=K_{p}+\frac{K_{d}}{s^{\alpha }}$$

where $K_{p}$ is the proportional coefficient and $K_{d}$ is derivative coefficient value of FOPD controller having α as fractional operator. Block diagram of using FOPD controller in simulations for closed-loop demand control is illustrated in Figure 4.

### 3.5. Super Twisting Sliding Mode Controller Proposed for Closed-Loop Elastic Demand Control

The STSMC algorithm is used for the control of the uncertainty of the linear plants to determine the robustness [53,54]. STSMC u(t) is given as follows:

(6) $$C_{STSMC}\left(t\right)=K_{1}e+K_{2}\left(\sqrt{\left|e\right|}\right)sign\left(e\right)+v\;$$

(7) $$v=K_{3}sign\left(e\right)$$

where, *e* is the error signal, $K_{1}$, $K_{2}$, $K_{3}$ are the parameters of the STSMC and sign is a function used to reduce the chattering phenomenon in STSMC. These parameters are tuned for the purpose to obtain optimal results.

One of the applications of STSMC is controlling nonlinear uncertain systems. STSMC has 3 coefficients; $K_{1}$ is the proportional control design constant, $K_{2}$ should be ranging between [0 1] and $K_{3}$ is the integral control design constant. As in this case, there is an inverse relationship between the price and demand of the system that is why the values of $K_{1}$ and $K_{3}$ should be negative, to gain energy balance.

## 4. Proposed System Model

**Figure 5:**
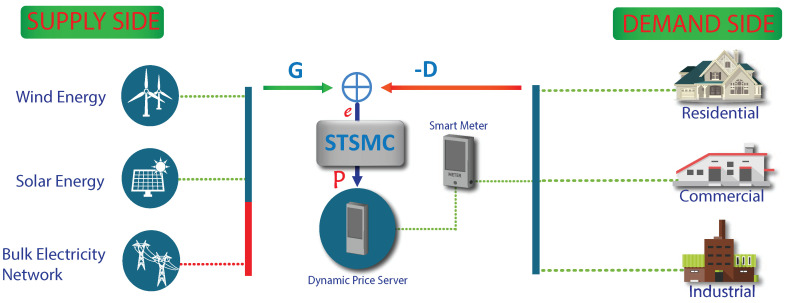
Schematic diagram for the proposed system model to control the elastic demand of consumers through dynamic pricing in a smart grid.

The purpose of this work is to set up tuning rules for achieving an a priori nominative settling time for planar systems. The feedback of the system is formed by using the STSMC to estimate one of the states. States which are estimated and measured, are then used by the STSMC for tuning in the presence of external disruption. We propose STSMC in this work because of its robustness and accuracy. The proposed STSMC technique narrows down the gap and the demand traces the generation profile in an optimal manner. This work is the continuation of our earlier conference paper [55]. With the increase in population, the energy demand has drastically increased. To cope with this increased electricity demand, the distribution system operator provides DR programs. In literature, various DR programs exist like price-based DR programs, and incentives-based DR programs to motivate consumers to take part in demand side management [5]. In this work, we use dynamic energy pricing which is one of the price-based DR programs. Energy must be supplied for operations in real-time in case there is a change in generation from the supply side. Therefore, we have to control the demand of consumers according to generation to ensure the supply of energy in intermittent conditions. The proposed model is shown in Figure 5. It comprises of the supply side, dynamic price generation server and DSLM.

**Figure 6:**
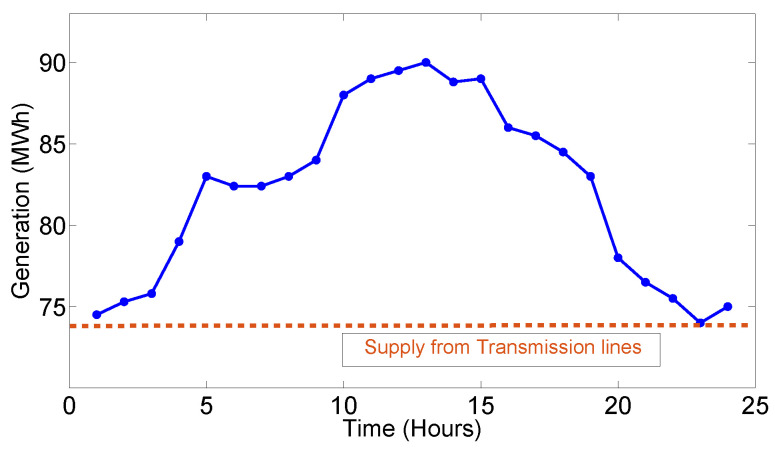
Renewable energy generation and supply from bulk utility supply data.

The supply for the proposed model is fluctuating renewable energy that will be supplied from a local renewable energy grid station with a utility bulk power supply which is used as a backup source. This bulk electricity from utility will be supplied when the generation from renewable energy is deficient. The renewable energy supply profile values are taken from [56] as shown in Figure 6, which is increased from kWh to MWh because the smart microgrid considered in this work is serving highly dense residential, commercial and industrial sector, where consumers demand is high and required more energy. In addition, generation values were increased to MWh level for fair evaluation of our proposed model and scenario built for the simulation purpose. Wind and solar energies have taken for the production of renewable energy at the local microgrid along with bulk utility supply from transmission lines. The dynamic price generation server comprises of the following main components;

- A power system that consists of generation, transmission lines and distribution system.
- A communication system between the supply side and the demand side. This communication is done through power lines, modems, routers, wireless technologies, etc.
- An STSMC which originates a price signal based on the mismatch between the instant demand and generation.

The error signal feeding in to STSMC can be expressed through Equation (8);

(8) e=G(t)−Do(t)

where *G(t)* is the instant generation and *Do(t)* is the instant demand of the consumer which feedback to the system again numerically given by the mismatch between the instant demand and the instant generation known as error (*e*) into the STSMC.

The following equation is taken as elastic DR model function with time constant τ of consumer load response [14].

(9) $$\tau \frac{dDo(t)}{dt}+Do\left(t\right)=D\left(p\right)\;$$

In Equation (9), *Do*(*t*) refers to the demand of consumers at any instant of time and *D*(*p*) refers to the price DR function of a market which is taken here as piece-wise-linear function. There is also a delay $L_{P}$ in broadcasting the price signal to the DSLM system’s agents at the demand side and its response to the price signal. Dynamic electricity pricing and the DR to it will make the demand of consumer easy to predict in smart grid for the short term.

**Figure 7:**
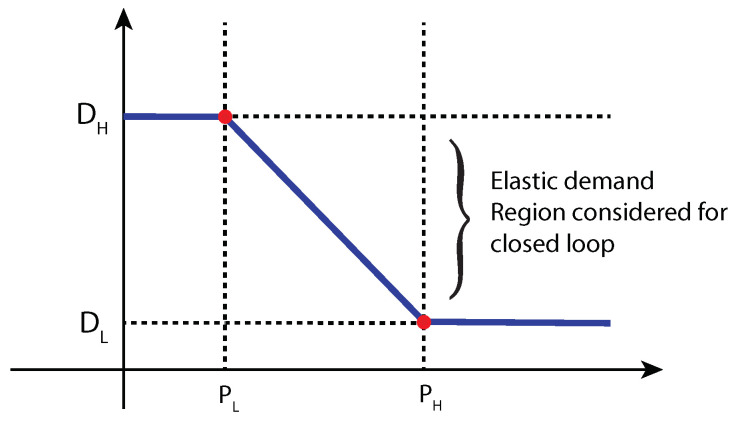
Parameters for demand elasticity function.

It is the basic principle of the market that when the price of something increases automatically, the demand of it decreases drastically. Based on this principle of the market, this whole system is designed for elastic demand control. The price demand model is designed in such a way that it has a high demand $D_{H}$ of a consumer which has to be provided and low demand $D_{L}$ that is provided from the utility bulk energy system. Price-based demand model consists of the following properties;

1. There are always limited consumers to which the electricity has to be supplied. We assume that for a low price $P_{L}$ of electricity there must be $D_{H}$ of the consumer. Therefore, DSLM shifts all the load of consumers to that point where electricity price is low. At ($P_{L}$, $D_{H}$) the demand elasticity deteriorates as defined in Figure 7.
2. As there are critical loads, electricity must be supplied to them even at a high price $P_{H}$. Therefore, the demand of consumers will be low at this point ($P_{H}$, $D_{L}$) and demand elasticity also deteriorates at this point.
3. In the range between $P_{H}$ and $P_{L}$, demand condition is elastic. When the pricing signal is between these two points, the demand of consumers will depend upon the pricing signal. The elasticity of demand in this region is determined as shown in Equation (10);

(10) $$ElasticDemand=\frac{LowDemand(DL)}{HighDemand(DH)}$$

In Equation (10), the elastic demand value ranges between 0 and 1. Value close to zero indicates that there is elasticity in the demand. Values close to 1 means that there is no elasticity in consumer demand. In [14], the following piece-wise-linear function for price-based DR is estimated by considering Figure 7;

(11) $$D\left(p\right)=\left\{\begin{array}{c} \;\;\;\;\;\;\;\;D_{H}\;\;\;\;\;\;\;p<0.35\\ \frac{\left(D_{H}-D_{L}\right)}{\left(P_{L}-P_{H}\right)}\left(p-P_{L}\right)\\ \;\;\;\;\;\;\;\;D_{L}\;\;\;\;\;\;\;\;\;p>P_{H} \end{array}\right.P_{L}\leq p\leq P_{H}\;$$

By taking Laplace transform of Equation (9), we obtain Equation (12) for the instant demand of the consumers which is composed of the elastic demand function and transfer function;

(12) $$Do\left(s\right)=D\left(p\right)\frac{1}{\tau s+1}\;$$

The price varying first-order dynamic equation for the overall demand of consumers’ response of electricity market models is the above equation. There are uncertain conditions sometimes at the demand side where the demand increases or decreases abruptly. In these cases, the grid can reduce uncertainty and make the DR of consumer easy to predict. In simulations, this uncertainty factor was added through generating random numbers in the overall DR to make the simulation more realistic and similar to operations taking place in smart grids. Figure 13 shows overall renewable energy generation profile.

## 5. Simulations and Discussion

**Figure 8:**
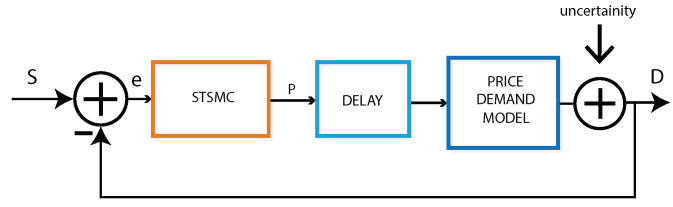
Simulink block diagram for the proposed model.

For simulations, MATLAB/Simulink is used for creating the overall environment for the proposed model and to implement the closed-loop elastic demand control. The block diagram of the simulations is shown in Figure 8. STSMC is used to adjust the prices of electricity in the local market. This controller has 3 control design coefficients and is configured to get the DR of the consumer. There are 3 gains of STSMC: K1, K2 and K3. K1 is the proportional control design, K2 should be between 0 and 1 and K3 is the integral control design constant.

### 5.1. Step Response Analysis

**Figure 9:**
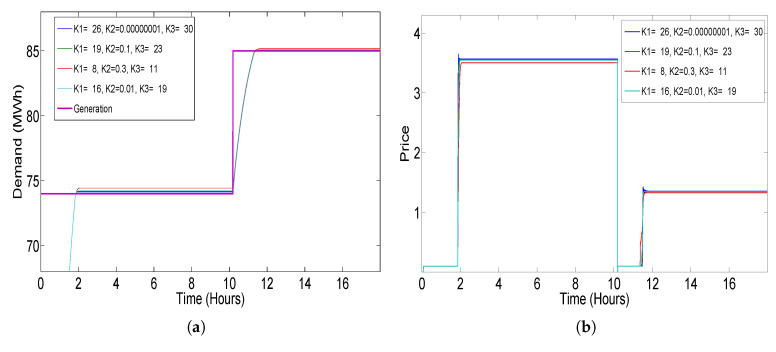
Demand and price response of the proposed model to step signal for various coefficients K1, K2 and K3 of STSMC: (**a**) demand response; (**b**) price response.

**Figure 10:**
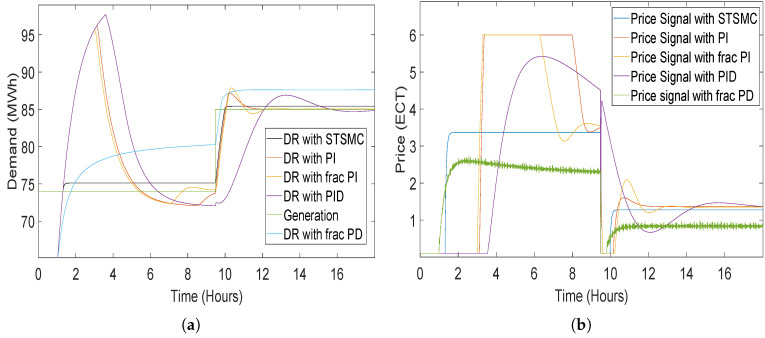
Demand and price response of the proposed model to step signal for classical and fractional order controllers: (**a**) demand response; (**b**) price response.

**Figure 11:**
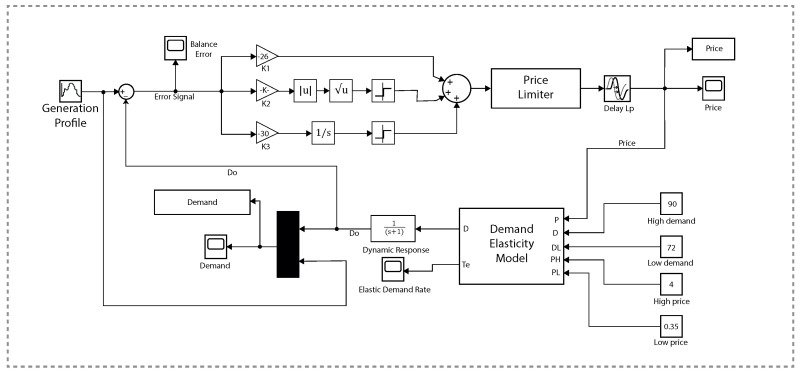
Simulink model formulated for the simulations of demand and price response of closed-loop elastic demand control by STSMC.

The step response of the closed-loop elastic demand control for energy market simulation is demonstrated in Figure 9 using various coefficients. Step response of the system is considered for the sharp rise and fall in the generation. For getting the DR of the system, whenever, there is a sharp rise or sharp fall in the generation which is due to fluctuating renewable energy, we have to get DR of the proposed model in the form of a step generation signal. For this purpose, the step generation signal is passed through the STSMC and we get the demand and price response through various coefficients of the STSMC. Figure 10 and Figure 11 show the block diagram of the simulation formulated for getting demand and price response from the various generation input to the closed-loop system. The MATLAB/Simulink simulation model consists of a delay $L_{P}$ = 36 s, piece-wise-linear price demand function, and time constant as τ = 1 h.

As a result of the inverse relationship between demand and price, the coefficients K1 and K3 of the STSMC are tuned negative. K2 will be between 0 and 1. Figure 9 shows the result of demand and price response of the system using various coefficient values of STSMC. Demand and price response, which we get through changing the coefficients of STSMC, which are according to our desired output, are used later on in the simulation environment which is created for the whole scenario shown in Figure 5. In Table 2, coefficient values used for PI, FOPI, PID, FOPD and STSMC controllers are presented. The coefficients’ values of PI, FOPI and PID controllers were taken from [14,43] and for STSMC, we have selected those coefficients’ values which give better response as shown in Figure 9.

For the response of demand and price to a step generation signal as an input using the STSMC, classical and fractional order controllers are shown in Figure 10. PI controller, which has gain coefficients $K_{P}$ and $K_{I}$, where $K_{P}$ is the proportional gain and $K_{I}$ is the integral gain of the PI controller. The coefficient values for PI controller are taken negatively because of the inverse proportion between demand and price of electricity [14]. Thus, values of $K_{P}$ = −1 and $K_{I}$ = −2 are taken. The results from the STSMC, classical and fractional order controllers mapped to a step generation signal validate that STSMC is more robust and accurate having lesser transient response, whereas other controllers overshoot at the start of the simulations.

### 5.2. Scenario: Demand and Price Response of the System

In this section, there is a scenario created for closed-loop elastic demand control for which the MATLAB/Simulink model is formulated. The block diagram for the simulations is shown in Figure 11. In the simulation scenario, the energy generation profile is composed of wind and solar energy from renewable energy generation local grid $Do_{MAX}$ as well as bulk utility supply from the transmission system ($T_{S}$) of the grid. Energy from ($T_{R}$) and ($T_{S}$) can be written as Equation (13);

(13) $$Do=T_{R}+T_{S}\;$$

where Do is the local demand and is considered the elastic demand. There is also a high demand ($D_{H}$) and a low demand ($D_{L}$) in a scenario for which the price response and DR are approximated through the piecewise linear function as mentioned in the Equation (11). The supply from the grid and renewable energy generation can be conceived as to make up for the electricity shortages when the demand of consumers is low. However, there will also be critical loads to which the electricity has to be supplied even in the inefficient hours when there is zero renewable energy generation from the local grid. Therefore, the minimum local demand can be planned as shown in Equation (14);

(14) $$Do_{MIN}\leq T_{R}+T_{S}\;$$

To avoid the excessive energy generation and to cut down the expenses of the production of energy, there is a limit which is equivalent to the maximum demand of the consumer that cannot be exceeded during the supply of energy. This can be represented as shown in Equation (15);

(15) $$T_{R}+T_{S}=Do_{MAX}\;$$

For the maximum efficiency and reliable utilisation of the renewable energy generation, the overall generation and minimum and maximum local demand of the consumer can be planned as shown in Equation (16);

(16) $$Do_{MIN}\leq T_{R}+T_{S}\leq Do_{MAX}\;$$

In Equation (11), for the piece-wise linear-demand function, the upper bound is denoted by $Do_{MAX}$ and the lower bound of the demand is considered as $Do_{MIN}$, respectively. Now, for the low demand or critical loads when there is no supply from renewable energy local grid, then the supply to the consumer can be planned as $Do_{MIN}$ = ($T_{S}$), to ensure uninterrupted supply to the consumer. For the continuous supply from the renewable energy local grid, the $Do_{MAX}$ of the consumer is planned as $Do_{MAX}$ = ($T_{R}$) +($T_{S}$). Now, to meet the requirement of the consumer and to cut down the additional expenses of the operational costs, the renewable energy generation for this scenario can be planned as in Equation (17);

(17) $$T_{R}=Do_{MAX}-Do_{MIN}\;$$

**Figure 12:**
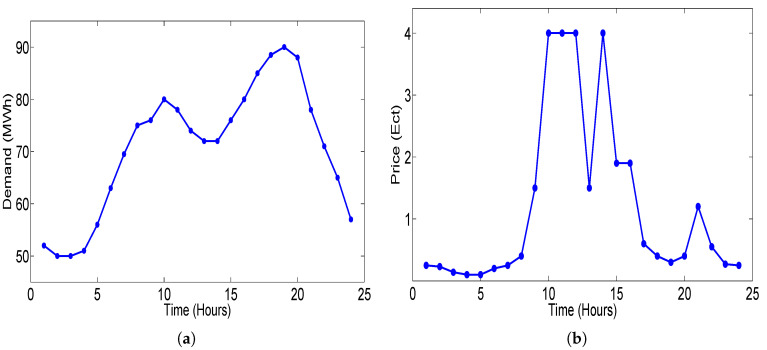
Demand and price data considered for making demand function: (**a**) hourly electricity demand; (**b**) hourly price of electricity.

Equation (17) refers to the renewable energy generation for the closed-loop elastic demand control. For the formulation of piece-wise-linear function for the closed-loop elastic demand model, the price of the electricity and demand of the consumer for 24 h is taken from [53] and has been scaled to MWh level and shown in Figure 12. The price and demand data are combined time independently and a data set was formed ($P_{i}$, $D_{i}$) for *i* = 1, 2, 3, ..., 24. When this data set is formed in [54], the values which are not in accordance with our piece-wise linear-demand function are excluded through Equation (18):

(18) $$\left(\frac{dD(t)}{dt}\right)\left(\frac{dp(t)}{dt}\right)<0\;$$

The elastic demand control of the system is estimated to price using a piece-wise-linear function. The demand function of the system is as follows in Equation (19):

(19) $$D\left(t\right)=\left\{\begin{array}{cc} 90 & p<0.35\\ -4.93p+91.72 & 0.35\leq p\leq 4\\ 72 & p>4\; \end{array}\right.$$

In Equation (12), for the high price of energy there will be low demand of the consumer and also for the low price the consumer will try to use much energy and the demand will be high. This piece-wise-linear function is formulated keeping in mind the general principle of energy market.

**Figure 13:**
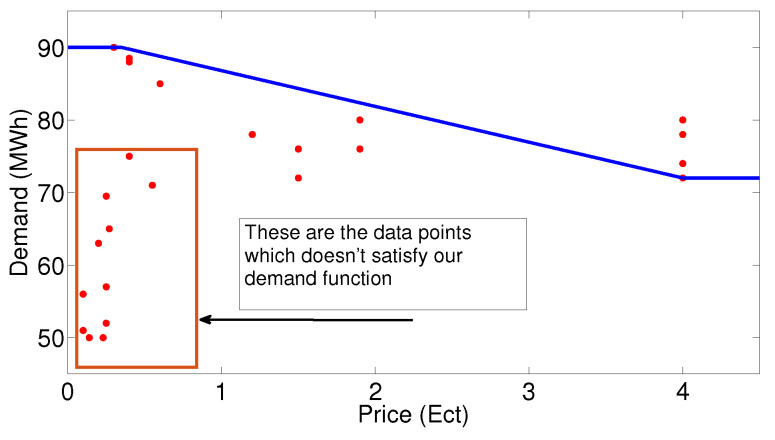
Piece-wise-linear function with Pi and Di.

This piece-wise demand function is designed for the closed-loop elastic DR and states that when the price of electricity is low, then there will be high demand of consumers and when the price of the electricity is high, then the demand of the consumer is low, depending on the price signal coming from the STSMC. The piece-wise-linear function formed for the price DR is shown in Figure 13.

Equation (19) is used as the price demand model in the overall simulation of the system. In Equation (19), the $Do_{MAX}$ = 90, which will be supplied to the consumer when the price signal $P_{L}$ is below or equal to 0.35 and for the price signal $P_{H}$ higher than 4, then the demand of the consumer will be $Do_{MIN}$ = 72 according to the piece-wise linear-demand function. The $T_{R}$ can be planned as $T_{R}$ = 90 MWh−72 MWh. As 72 MWh of energy is supplied from the transmission lines and the overall demand is 90 MWh, so the $T_{R}$ can be planned as 18 MWh which is produced at local microgrid and numerically shown in Equation (15). The overall DR of the system is represented numerically in Equation (12).

#### 5.2.1. Closed-Loop Elastic Demand Control Using PI Controller

**Figure 14:**
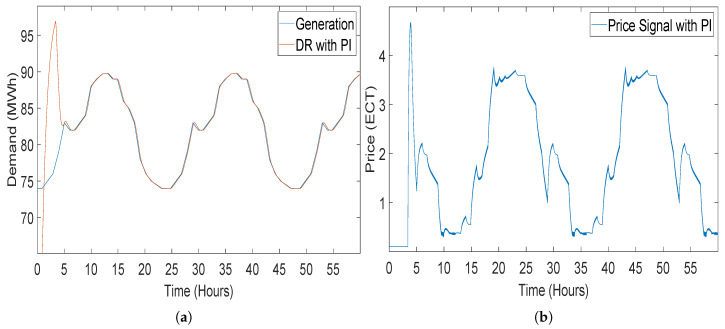
Closed-loop elastic demand control using a proportional integral (PI) controller having coefficient values $K_{p}$ = −1 and $K_{p}$ = −2: (**a**) demand response; (**b**) price response.

PI controllers have 2 control coefficients $K_{p}$ and $K_{i}$. The values for the coefficients were given manually and both the values were kept negative because of the inverse relation between demand and price of energy. Figure 14a shows 60 h of simulation result, in which generation is being traced by the demand of consumer. Figure 14b shows the price of energy which is regulated to the smart meters at the consumer end through which DSLM adjust the load of consumer and increases and decreases the demand according to the pricing signal regulated by the PI controller.

Getting response for the system by using PI controller, there is a strong tendency of getting an overshoot at the start [14]. Although the demand traced the generation effectively, the tracing time is greater as compared to the STSMC. There is an overshoot at the start of the simulation before generation was traced by the demand. This overshoot at the start can blackout at the consumer end, because of the unavailability of the energy at the start. The DR of the system using the PI controller before the tracing of demand is as high as 90 MWh and the generation in this region is not this much, which means lack of power at the consumer end during the overshoot time which is 5.5 h [14].

#### 5.2.2. Closed-Loop Elastic Demand Control Using Fractional Order PI (FOPI) Controller

**Figure 15:**
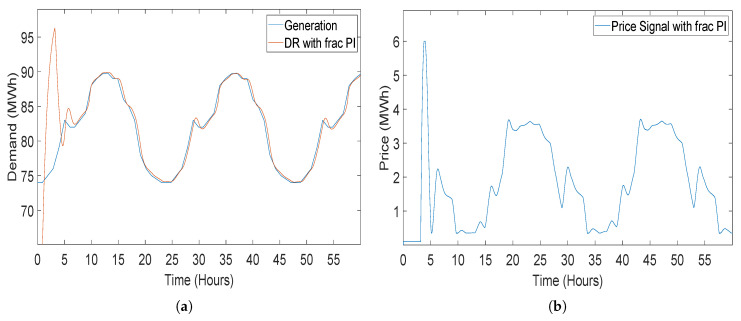
Closed-loop elastic demand control using a fractional order proportional integral (FOPI) controller having coefficient values $K_{p}$ = −1, $K_{p}$ = −2 and λ = 0.8: (**a**) demand response; (**b**) price response.

A PI controller with additional fraction order operator called λ makes a fractional order PI controller. This FOPI controller is presented for a closed-loop elastic demand control which was a model based system [43]. The DR of the current system by using FOPI gives an overshoot at the start of the simulation even more than the demand controlled by PI controller. Demand of consumer takes longer tracing time which also results in blackout or load-shedding at the consumer end. Figure 15a demonstrates the generation being traced by demand of consumer using FOPI controller. The demand traced down generation after overshoot for the first 7 h. Figure 15b shows the pricing signal which is regulated to the smart meters at the consumers’ end for elastic demand control by FOPI.

Same as the PI controller, the coefficient values for the FOPI are kept negative because of inverse relation between demand and price, whereas the value for λ is taken as 0.8.

#### 5.2.3. Closed-Loop Elastic Demand Control Using PID Controller

**Figure 16:**
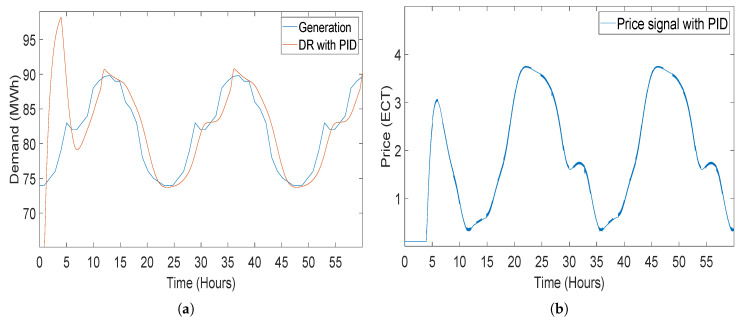
Closed-loop elastic demand control using a proportional integral derivative (PID) controller having coefficient values $K_{p}$ = −1, $K_{p}$ = −2 and $K_{d}$ = −3: (**a**) demand response; (**b**) price response.

Proportional integral derivative (PID) is the widely used classical controller. PID controllers have 3 control coefficients $K_{p}$, $K_{i}$ and $K_{d}$, the values of which are kept negative. PID is presented for the closed-loop elastic demand control which is effectively controlled by the PID but with an overshoot and large error between the generation and demand of consumer [43]. The demand followed the generation but with an extra large tracing time as compared with demand controlled by PI and FOPI. Both FOPI and PI outperform the PID controller in tracing the generation. Closed-loop elastic demand control using PID controller having coefficients is shown in Figure 16.

#### 5.2.4. Closed-Loop Elastic Demand Control Using FOPD Controller

**Figure 17:**
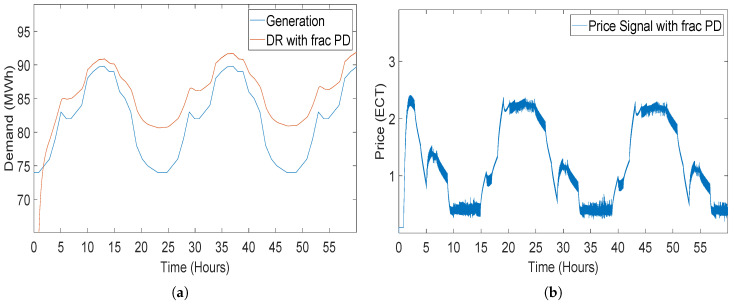
Closed-loop elastic demand control using a fractional order proportional derivative (FOPD) controller having coefficient values $K_{p}$ = −0.2, $K_{p}$ = −0.3 and α = 0.3: (**a**) demand response; (**b**) price response.

When the simulation of the closed-loop elastic demand using the FOPD controller starts, the demand starts tracing the generation with overshooting at the start of the simulation. Although generation started to trace generation, the gap between the generation and demand of consumer can be clearly seen in Figure 17. In Figure 17b, the pricing signal provided by FOPD controller to the demand model is demonstrated. The pricing signal provided by the FOPD controller is less than all the controllers but the error in tracing the generation by demand is very high as compared to other controllers (STSMC, PI, PID and FOPI), which were deployed in the same system for elastic demand control.

#### 5.2.5. Closed-Loop Elastic Demand Control Using STSMC Controller

When the simulation starts, the demand of the consumer is controlled successfully with STSMC by continuously providing a price signal to the elastic demand model. The demand started tracing the renewable energy generation at about 1.8 h after starting the simulation. The tracing time of generation by demand is the lowest among the controllers. i.e., PI, FOPI, PID and FOPD. The demand of consumer traced the renewable energy generation to the end of the simulation. The results of the simulation show that closed-loop elastic demand control with STSMC can successfully trace renewable energy generation without overshooting at the start of the simulation. The demand of the system also does not overshoot at the start of the simulation as in [14,43] by using the classical and fractional order controllers. In order to reduce price volatility, a price limiter function is added next to STSMC which reduces the dynamic price volatility due to uncertainty in the DR. Equation (20) shows the function for price limiter. The upper bound in Equation (20) limits the pricing signal to not feed a pricing signal lower than 0 or negative pricing signal. In addition, the lower bound is for controlling the price to not exceed a specified limit.

(20) $$PriceLimter=\left\{\begin{array}{c} 0.1\;\;\;\;\;\;\;\;\;\;p<0\;\\ p\\ 12\;\;\;\;\;\;\;\;\;\;p>12\; \end{array}\right.\;0.1\leq p\leq 12\;\;$$

The results of the simulation show that closed-loop elastic demand control in conjunction with STSMC can successfully trace the renewable energy generation without overshooting at the start of the simulation.

**Figure 18:**
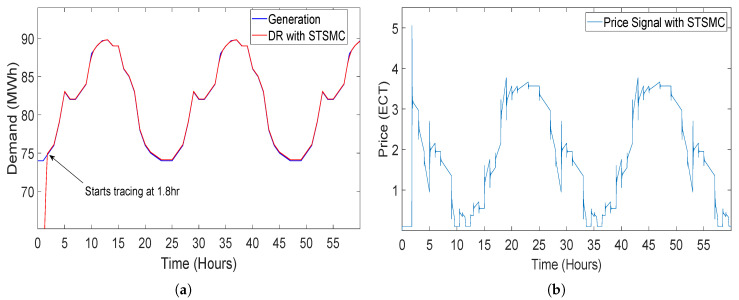
Closed-loop elastic demand control using super twisting sliding mode (STSMC) controller having coefficient values $K_{1}$ = −26, $K_{2}$= 0.0000001 and $K_{3}$ = −30: (**a**) demand response; (**b**) price response.

**Figure 19:**
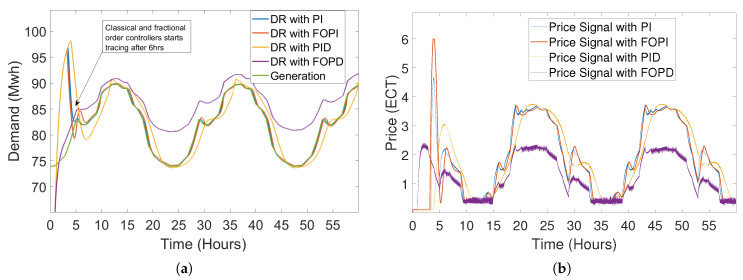
Closed-loop elastic demand control using PI, FOPI, PID and FOPD controllers: (**a**) demand response; (**b**) price response.

This whole scenario is discussed in [14], where the closed-loop elastic demand of the consumer is controlled by the PI controller. Moreover, the demand started tracing generation after 5.5 h which is a long time as compared to the tracing time of the STSMC. In our paper, we have deployed STSMC, which is a more robust, accurate and advanced algorithm/controller than the classical controllers and fractional order controllers. Figure 18 shows the DR of the elastic demand control by STSMC. In Figure 19, a clear difference can be seen in the graph where the classical and fractional order controllers overshoots at the start of the simulations, but STSMC started tracing the generation 1.8 h after the start of the simulations. The demand starts from the zero initial value and takes 1.8 h to reach generation, after that the demand does not overshoot and starts tracing generation. Whereas by using PI, FOPI and PID controllers, demand sharply increases, which results in demand overshoot at the beginning of simulations for 6 h. Although the FOPD does not overshoot at the start unlike the other controllers, error in tracing the generation is very high among these controllers. Moreover, the difference between the pricing signal provided by the classical and fractional order controllers can also be seen in Figure 19b. The STSMC provides pricing signal directly at 1.8 h after the start of simulations to quickly control the demand of consumers but the classical and fractional order controller lags in providing the pricing signal, thus results in overshooting of demand.

STSMC outperforms PI, FOPI, PID and FOPD controllers in tracing the generation by the demand of consumer in a renewable energy integrated microgrid. The values of DR of STSMC in comparison with PI, FOPI, PID and FOPD are shown in Table 3, which shows the DR of closed-loop elastic demand controllers for 60 h. Table 4 shows the data of error in tracing the generation for every two hours of simulations for classical, fractional order and STSMC. It can be clearly seen from Table 4 that STSMC outperforms classical as well as fractional order controllers. The coefficient values of the PI controller were kept the same which are used for demand control in [14]. In addition, the coefficient values for the FOPI and PID controller were kept the same as in [43]. The error between the generation and the DR of the deployed controllers in closed-loop elastic demand control simulations is shown in Table 3.

**Figure 20:**
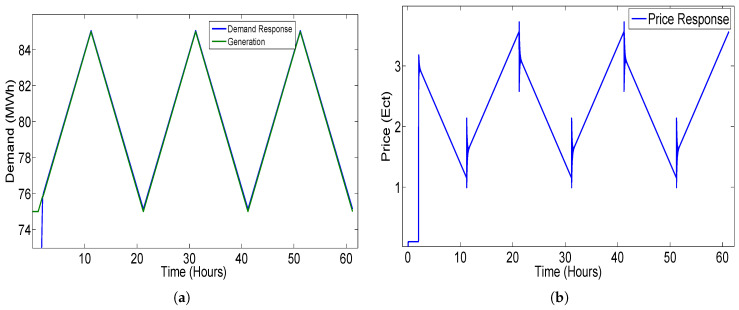
Closed-loop elastic demand control using super twisting sliding mode (STSMC) controller with triangular input having coefficient values $K_{1}$ = −26, $K_{2}$= 0.001 and $K_{3}$ = −30: (**a**) demand response; (**b**) price response.

The demand and price response of the closed-loop elastic demand-control model for the triangular waveform as input is shown in Figure 20.

## 6. Conclusions

This paper employs elastic demand control of a renewable generation integrated microgrid by a closed−loop STSMC. This STSMC regulates the price signal of the DSLM system which plays a major role in the automation of demand control and also making demand more predictable and deterministic. Price signal from STSMC feeds into the DSLM installed in smart meters at consumers’ premises through which the balance of generation and demand of the microgrid is accomplished via closed-loop STSMC. In this paper, the renewable energy integrated microgrid scenario is discussed through which the closed-loop elastic demand of the system is controlled by tuning the coefficients of the STSMC. For simulations, the whole scenario environment is simulated in MATLAB/Simulink. The price-based demand model for the current scenario is obtained from the hourly price and demand data. In addition, conditions of high demand and low demand of the consumer were discussed for electricity import from the bulk utility supply. This was added in simulations to cut down the additional expenses and operational cost in the smart grid. The process of broadcasting the price to the DSLM agents takes time, in order to show effectiveness of the method in the cases of demand uncertainty and system delays. We used price signal-broadcast delay for making simulations for scenario more realistic. System delay was added after STSMC in the system which is equal to 36 s. We observed from the simulations that price-based elastic demand control by STSMC can exhibit the outstanding performance of tracing of renewable energy integrated microgrid generation. Dynamic energy pricing is a key figure in the smart grid to control the demand of consumers in renewable energy market management. Our findings shows that the STSMC, very quickly after just 1.8 h, starts tracing the generation. The classical and fractional order controller overshoots and follows to trace the generation after 6 h which is significantly higher as compared with STSMC. Thus, our proposed method outperforms the existing methods.

## Figures and Tables

**Table 1:** Summary of related work in terms of techniques, model, objectives, results and limitations.

References	Techniques	Models	Objectives	Results	Limitations
[19]	MILP	Flexible DR load model	Flexible load control based on DR	Efficiency, power system adjustment capability and safety of power grid operation enhanced	Only flexible loads are considered
[20]	Master controller	Advanced metering infrastructure with HEMS	To better manage the energy at the consumer side	Based on the categorisation of DR programs proper load management is accomplished	User discomfort
[21]	BBSA and BPSO	HEMS	To reduce energy cost, electricity bill and peak load	BBSA gives better results than BPSO	PAR is not considered
[22]	MOPSO and BILP	Intelligently responsive HEMS	To reduce cost and carbon emission	Electricity cost is reduced by 10.25% and also carbon emission is reduced	Carbon emission is reduced and operating cost is increased
[23]	MPC	Hybrid system under TOU with power selling	A battery storage system with solar panels to reduce cost in peak hours	Batteries provide power in peak hours and reduce the monthly cost	PAR consumer comfort is not considered
[24]	LSHS	Intelligent energy management system (IEMS)	Load scheduling at consumer end with increasing efficiency	The system is optimised	System complexity and user discomfort increased
[27]	MILP	HEMS	To reduce consumer inconvenience caused by DR programs	Increased energy efficiency within domestic environment	Did not considered uncertainty in DR
[28]	GA	DSLM using load-shifting concept	To reduce overall peak load demand and operational cost	Reduction in power demand and cost of the utilities	Cost reduced but ignored consumers’ comfort
[29]	EMA and Fuzzy logic controller	HEMS	Electricity and fuel cost reduction with increasing efficiency and lifetime of fuel cell	Energy efficiency of solar, wind and fuel cell increased	Did not considered carbon emission reduction
[30]	MPC	Dynamic optimisation- based DR scheduling framework and low-order Hammerstein Wiener model	Energy management and electricity cost reduction	Electricity cost reduced	Considered price forecast but didn’t considered DR uncertainty
[31]	MPC	BESS and HVAC Scheduling	To optimally use battery storage	Electricity cost reduced	PAR and carbon emission not considered
[33,34,35]	MMIGA, CBI	Persuasive smart energy management system (PSEMS)	Prediction of consumers’ demand in SG	With closed-loop DR programs, demand becomes more deterministic and predictable	Uncertainty in demand is not considered
[36]	PID controller	Dynamic demand responsive generation management	To decrease the electricity bills of consumers and operational cost	Operational and electricity bill reduced	Electricity cost reduced and user discomfort increased
[37]	MILP	Price-based HEMS	Optimise the energy consumption by scheduling of appliances in smart home	Electricity cost reduced	System complexity Increased

**Table 2:** Coefficients and fractional operator values for different controller used for all simulations.

PI		FOPI			PID			FOPD			STSMC		
$K_{P}$	$K_{I}$	$K_{P}$	$K_{I}$	λ	$K_{P}$	$K_{I}$	$K_{d}$	$K_{P}$	$K_{d}$	α	$K_{1}$	$K_{2}$	$K_{3}$
−1	−2	−0.1	−1.2	0.8	−0.01	−0.2	−0.01	−0.2	−0.3	0.3	−26	0.001	−30

**Table 3:** Comparison DR of the closed-loop STSMC with PI [14], FOPI [43], PID [43] and FOPI demand response in tracing the generation.

DR of Closed-Loop Elastic Demand Control by Different Controllers.						
**Time (hours)**	**Generation (MW)**	**DR (PI)**	**DR (FOPI)**	**DR (PID)**	**DR (FOPD)**	**DR (STSMC)**
2	75.11	87.91	87.91	74.96	76.08	76.10
4	79.12	87.47	84.56	98.20	81.59	79.71
6	82.04	82.29	84.39	81.64	84.92	82.08
8	83.11	82.98	83.44	80.24	85.71	83.05
10	88.03	87.82	88.01	84.52	89.30	87.50
12	89.64	89.63	89.77	90.66	90.72	89.61
14	89.08	89.11	89.37	89.47	90.35	89.22
16	85.96	86.25	86.88	88.36	88.39	86.06
18	83.06	83.29	83.79	85.15	86.43	83.08
20	76.34	76.43	76.50	79.86	82.10	76.13
22	74.57	74.59	74.84	74.64	80.93	74.63
24	74.01	73.98	74.14	73.70	80.68	74.13
26	75.23	75.11	75.11	74.25	81.43	75.11
28	79.56	79.12	78.77	76.37	84.06	79.04
30	82.11	82.13	82.49	81.59	86.23	82.09
32	83.24	83.12	83.05	83.09	86.75	83.04
34	88.15	88.02	87.90	84.51	90.36	87.55
36	89.70	89.69	89.59	90.40	91.64	89.60
38	89.01	89.08	89.16	89.52	90.97	89.03
40	85.99	86.36	86.90	88.36	88.94	86.09
42	83.14	83.36	83.76	85.20	86.87	83.10
44	76.40	76.49	76.47	79.99	82.49	76.15
46	74.59	74.61	74.79	74.69	81.24	74.64
48	74.01	73.98	74.08	73.70	80.95	74.13
50	75.19	75.06	75.02	74.23	81.65	75.10
52	79.44	78.99	78.60	76.30	84.19	79.01
54	82.01	82.18	82.56	81.48	86.45	82.10
56	83.21	83.08	82.98	83.09	86.91	83.03
58	88.12	87.96	87.81	84.45	90.56	87.53
60	89.67	89.66	89.53	90.27	91.86	89.60	

**Table 4:** Error comparison in tracing the generation by demand of consumer using PI [14], FOPI [43], PID [43], FOPD and STSMC.

Time (hours)	Generation (MW)	PI	FOPI	PID	FOPD	STSMC
2	75.11	−12.8000	−12.8000	0.1500	−0.9700	−0.9900
4	79.12	−8.3500	−5.4400	−19.0800	−2.4700	−0.5900
6	82.04	−0.2500	−2.3500	0.4000	−2.8800	−0.0400
8	83.11	0.1300	−0.3300	2.8700	−2.6000	0.0600
10	88.03	0.2100	0.0200	3.5100	−1.2700	0.5300
12	89.64	0.0100	−0.1300	−1.0200	−1.0800	0.0300
14	89.08	−0.0300	−0.2900	−0.3900	−1.2700	−0.1400
16	85.96	−0.2900	−0.9200	−2.4000	−2.4300	−0.1000
18	83.06	−0.2300	−0.7300	−2.0900	−3.3700	−0.0200
20	76.34	−0.0900	−0.1600	−3.5200	−5.7600	0.2100
22	74.57	−0.0200	−0.2700	−0.0700	−6.3600	−0.0600
24	74.01	0.0300	−0.1300	0.3100	−6.6700	−0.1200
26	75.23	0.1200	0.1200	0.9800	−6.2000	0.1200
28	79.56	0.4400	0.7900	3.1900	−4.5000	0.5200
30	82.11	−0.0200	−0.3800	0.5200	−4.1200	0.0200
32	83.24	0.1200	0.1900	0.1500	−3.5100	0.2000
34	88.15	0.1300	0.2500	3.6400	−2.2100	0.6000
36	89.70	0.0100	0.1100	−0.7000	−1.9400	0.1000
38	89.01	−0.0700	−0.1500	−0.5100	−1.9600	−0.0200
40	85.99	−0.3700	−0.9100	−2.3700	−2.9500	−0.1000
42	83.14	−0.2200	−0.6200	−2.0600	−3.7300	0.0400
44	76.40	−0.0900	−0.0700	−3.5900	−6.0900	0.2500
46	74.59	−0.0200	−0.2000	−0.1000	−6.6500	−0.0500
48	74.01	0.0300	−0.0700	0.0600	−6.9400	−0.1200
50	75.19	0.1300	0.1700	0.9600	−6.4600	0.0900
52	79.44	0.4500	0.8400	3.1400	−4.7500	0.4300
54	82.01	−0.1700	−0.5500	0.5300	−4.4400	−0.0900
56	83.21	0.1300	0.2300	0.1200	−3.7000	0.1800
58	88.12	0.1600	0.3100	3.6700	−2.4400	0.5900
60	89.67	0.0100	0.1400	−0.6000	−2.1900	0.0700

## Abbreviations

**Symbols**	**Abbreviations**
τ	Time constant (h)
λ	Fractional operator in FOPI controller
α	Fractional operator in FOPD controller
*C(t)*	Controller function
*Do*	Local demand (MWh)
$Do_{MIN}$	Local minimum demand (MWh)
$Do_{MAX}$	Local maximum demand (MWh)
*Do(t)*	Instant demand (MWh)
*D(p)*	Piece-wise-price demand function
$D_{L}$	Low demand (MWh)
$D_{H}$	High demand (MWh)
*DSLM*	Demand side load management
*DR*	Demand response
*e*	Error (Mismatch between generation and demand
*FOPI*	Fraction order proportional integral controller
*FOPD*	Fractional order proportional derivative controller
*G(t)*	Overall instant generation (MWh)
$K_{p}$	Proportional coefficient
$K_{i}$	Integral coefficient
$K_{d}$	Derivative coefficient
$K_{i}$	Integral coefficient
$L_{P}$	Delay (sec)
*p*	Pricing signal (ECT)
*PI*	Proportional integral
*PID*	Proportional integral derivative
$P_{L}$	Low price (ECT)
$P_{H}$	High price (ECT)
*STSMC*	Super twisting sliding mode controller
*SMC*	Sliding mode controller
$T_{R}$	Transmission from local renewable energy system (MWh)
$T_{S}$	Transmission from bulk utility supply (MWh)
